# Comparative study of a novel application of automated HR HPV assay and stability in a previously untested Preservative media

**DOI:** 10.1016/j.pvr.2017.06.001

**Published:** 2017-06-04

**Authors:** Mike E. Morel, Simon E. McBride, Maria P. Gomez

**Affiliations:** Lab21 Ltd., Park House, Winship Road, Cambridge CB24 6BQ, United Kingdom

**Keywords:** Realtime HPV, Abbott RealTime High Risk HPV assay, CN, cycle number, HPV, Human Papilloma Virus, NHQ, Novaprep^®^ HQ+ orange, NHQ-PT, Novaprep^®^ HQ+ orange with pretreatment, ATB, Abbott Transport Buffer, STM, Digene Sample Transport Buffer. PS, Preservcyt. SP, Surepath, HPV, Preservative, Sample stability, Automated HR HPV assay

## Abstract

**Background:**

The suitability and stability of cervical cells in Novaprep media (NHQ) for certain HPV assays is unknown.

**Methods:**

We evaluated the accuracy of an automated HPV assay (Abbott RealTime HR HPV) for cervical cells prepared in NHQ and NHQ with a pre-treatment to mimic a worst case clinical use, compared to the assay manufacturers media; repeatability and reproducibility of HPV results and the stability of detectable HPV in NHQ over time compared to CE marked liquid based cytology preservatives. Cell lines were used to simulate patient samples.

**Results:**

Cells stored in NHQ produced accurate, repeatable and reproducible results. Stability in NHQ was comparable to the best performing LBC, with at least 7 months’ stability at 18–25 °C, 2–8 °C, −20 °C and −80 °C; and at least 3 months’ stability at 40 °C. Similar results were obtained for pre-treated NHQ except only 3.5 months’ stability at 18–25 °C. Cell line samples in all media and concentrations tested were detected appropriately by the assay.

**Conclusions:**

Based on this first stage validation analytical study, cervical cells stored in NHQ are suitable for the Realtime HPV assay. There should be no reservations for inclusion of NHQ in any further validation and clinical performance evaluation of this assay.

## Highlights

• Performance comparison of storage media for use with automated HR HPV assays.
• Cell stability demonstrated for up to 120 days at room temperature in Novaprep vials.
• Analytical performance assessment of HPV detection in Novacyt vials when used with an automated HR HPV assay.

## 1 Background

Human papilloma virus (HPV) is one of the most common sexually transmitted infections. Persistent HPV infection may lead to development of cancer and, in women, high risk HPV (HR HPV) is a prerequisite for the development of almost all types of cervical cancer. Testing for HR HPV has become integral to screening strategies, most of which combine HR HPV testing with cytological evaluation [1]. Of all known HPV genotypes, HPV16 or HPV18 cause approximately 70% of invasive cervical cancer cases worldwide, and are associated with higher risk of disease progression compared to other HR HPV genotypes [2], [3].

Various clinically validated molecular diagnostic tests to detect HR HPV exist, one of which is the Abbott RealTime High Risk HPV (RT HR HPV) test [4], [5], [6], a qualitative test utilizing Polymerase Chain Reaction (PCR) and probe hybridization to detect 14 HPV types in one assay. The test specifically identifies HPV16 and HPV18 whilst detecting other HPV types (31, 33, 35, 39, 45, 51, 52, 56, 58, 59, 66 and 68).

Specimens validated and CE-marked for use with the Realtime HPV test currently include cervical cells collected in ThinPrep Preservcyt Solution (PS), Surepath Preservative Fluid (SP), and the Abbott Transport Buffer (ATB).

Digene Specimen Transport Medium (STM) has also been shown to be compatible with the Realtime HPV test in a clinical validation study [7].

Currently ATB and STM are only intended for collection of samples for HPV testing, whereas PS and SP are Liquid Based Cytology (LBC) media primarily validated for cytology.

The ability to use the same sample for cytology and HPV testing offers significant advantages, it removes the requirement for two specimens in cervical cancer screening programs using cytology and HR HPV testing.

Novaprep® HQ+ orange (NHQ) is a LBC sample collection and storage device, for automated processing of cells onto cytology slides for cervical cancer screening by Novaprep Processor Systems® (NPS) and Novaprep Dragon Fly (NDF). Alongside PS, SP and STM, NHQ has demonstrated its suitability for collection and storage of cervical cells for a number clinically validated HR HPV tests including Hybrid Capture 2 (HC2) [8] and Cobas 4800 HPV [9]. However, suitability of cervical cell samples prepared in NHQ for the Abbott RT HR HPV test is currently unknown.

Cytology laboratories routinely treat cervical LBC specimens that are heavily contaminated with blood, mucus, inflammatory cells, or cellular debris with glacial acetic acid (GAA) to lyse red blood cells and facilitate assessment [10], [11]. However, the effect of GAA treatment of Preservcyt on the performance of HPV tests is controversial, at least one study for the Cervista HPV test showed that it increases false negative results [11], [12] and others for the HC2 and Abbott RT HR HPV tests showed little if any effect [13], [14]. Pretreatment of contaminated samples rich with blood cells, mucus or inflammatory clusters is also recommended by the NPS® manufacturer. Instead of GAA, specific manufacturer recommended wash procedures may be used. The potential impact of pretreatment on the performance of any HPV assays using NHQ samples is currently unknown.

The study described here is an analytical evaluation of the suitability of NHQ media, and pre-treated NHQ (NHQ-PT) for storage of human cervical cancer cells for testing with the Realtime HPV test.

## 2 Methods

### 2.1 Cell lines

Human cell lines containing HPV genome or negative for HPV were used to mimic cervical cell patient samples: SiHa human cervical squamous cell carcinoma cell line (ATCC® HTB-35™) has an integrated HPV16 genome (1–2 copies per cell); CaSki human epidermoid cervical carcinoma cell line(ATCC® CRM-CRL-1550™) also has an integrated HPV16 genome (about 600 copies per cell). C-33A human cervical carcinoma cell line (ATCC® HTB-31™) has no HPV genome. All cells were cultured by BioSynergy Europe Limited, Cambridge; Cells were counted, pelleted and re-suspended in the desired preservation media to the required concentration of cells and HPV genomic copies. For the purposes of this study, SiHa cells were estimated to have 2 copies, and CaSki cells were estimated to have 600 copies of HPV16 DNA per cell.

#### 2.1.1 Cervical cell storage media

Novaprep HQ+ orange (NHQ, Ref. NOV001N, Novacyt, Vélizy-Villacoublay, France);

Abbott Transport Buffer (ATB, List No. AB805, Abbott, Weisbaden, Germany);

Preservcyt Solution (PS, Ref. 0234005, Hologic, Wiesbaden-Nordenstadt, Germany);

Surepath vial (SP, Item No. 490527, Becton Dickinson, Temse, Belgium) are transport and preservation solutions for gynaecological and other cellular samples for cytological and molecular applications.

A NHQ variant designed to mimic pre-treated samples prepared according to manufacturer's instructions NHQ-PT includes added:

Dithiothreitol (Product No. 19733320, MOLEKULA, Gillingham, UK);

Ethylenediaminetetraacetic acid (Product No. E-6758, Sigma-Aldrich, Saint Louis, USA);
KH_2_PO_4_ (Cat. No. 1.06585, Merck Production Chemicals, Darmstadt, Germany);
Na_2_HPO_4_ (Cat. No. 26922.295, VWR Chemicals, Leuven, Belgium).

### 2.2 DNA extraction and HPV testing procedure

Cells in storage media (400 μl) were extracted and tested, according to manufacturer's instructions, on the Abbott m2000 system comprising the m2000sp robotic platform (No. 9K20) and the m2000rt real-time PCR machine (No. 9K25), using the Abbott RealTi*m*e High Risk HPV Amplification Kit (No. 2N09-92) and the Abbott RealTi*m*e High Risk HPV Control Kit (No. 2N09-80, ABBOTT, Max-Planck-Ring 2, 65205 Wiesbaden, Germany).

### 2.3 Accuracy of HPV test results for cells stored in NHQ

The accuracy of HPV detection in DNA from cells in NHQ compared to cells in ATB, which is CE-marked for storage of cervical cells for testing by the Realtime HPV assay, was evaluated by testing a range of CaSki, SiHa and C-33A cell numbers in NHQ or ATB in duplicate.

### 2.4 Repeatability and reproducibility of HPV test results for cells stored in NHQ

Within-assay repeatability of the HPV results for cells in NHQ was evaluated by testing three different concentrations of SiHa cells, each concentration repeated 14 times in a single run. Reproducibility of results for cells in NHQ was assessed by testing six samples of the same concentration of SiHa cells, prepared in three different lots of NHQ and NHQ-PT, 14 times in three different runs on two different days by a single operator.

### 2.5 Stability of HPV positive cells stored in NHQ for HPV testing

Stability of HPV16 DNA in SiHa cells for HPV testing after storage at −80 °C, −20 °C, 5 °C, 15–30 °C or 40 °C was assessed, comparing the stability between three lots of NHQ, three lots of NHQ-PT, one lot of PS and one lot of SP. A single sample was tested at each time point for each media lot.

The concentration of HPV DNA for the stability tests was chosen to replicate the lowest concentration used in the repeatability study. Although the level is well above the limit of detection for this assay the strength gives enough scope to examine signal stability over time.

## 3 Results

**Fig. 1:**
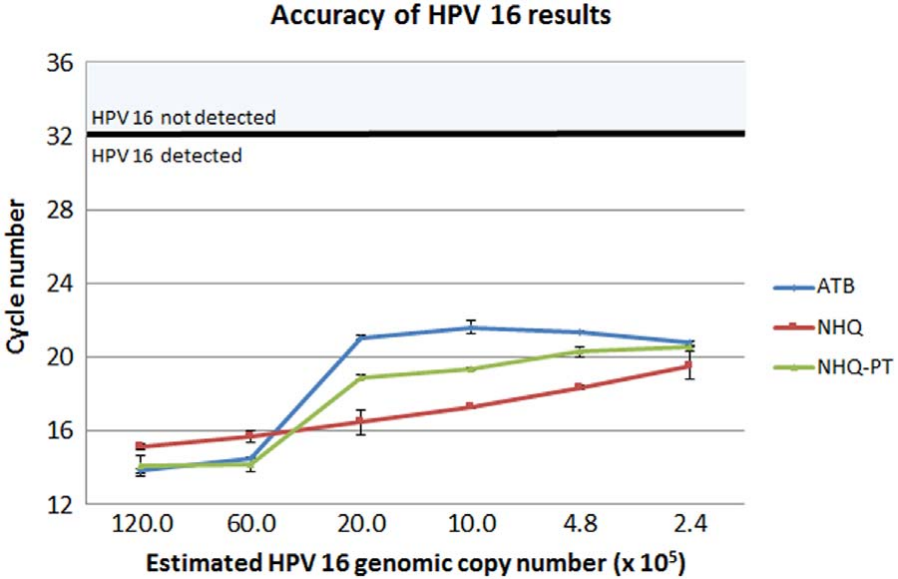
Accuracy of HPV 16 results using HPV 16 positive Ca Ski and SiHa cells, and HPV negative C-33 A cells in ATB, NHQ or NHQ-PT media as samples. Average CN value for two duplicates is shown with standard deviation error bars. Samples in which HPV 16 is detected have CN of 32 or below. CN, cycle number; ATB, Abbott Transport Buffer; NHQ, Novaprep HQ+ Orange; NHQ-PT, Novaprep HQ+ Orange (pre-treated).

Accuracy of HPV16 DNA detection from cells in NHQ and NHQ-PT was compared to cells in ATB using a range of cell and HPV16 copy numbers (Fig. 1). There was some variation in the Cycle Number (CN) values between different media samples with the same HPV16 copy number, which was most pronounced for samples containing an estimated 4.8−20 × 10^5^ HPV16 copies per assay. Within this range, NHQ samples gave CN values up to 5 cycles lower than ATB. Even though the assay is not quantitative, the NHQ CN results were more linear than those for ATB.

The CN results for samples in NHQ-PT were also lower compared to ATB in samples within an estimated 4.8−20×10^5^ HPV16 copies per assay, but higher than for samples in NHQ. The CN values and shape of the curve were closer to samples prepared in ATB than those prepared in NHQ. As the samples were tested in duplicate there are insufficient data to determine the reason for the shape of the curve and absence of linearity. This non-linearity could be due to a number of causes for example sample preparation, aliquoting, dilution effects, re-suspension effects, however all samples in NHQ, NHQ-PT and ATB were prepared at the same time from the same cell line stocks and the shape in NHQ is more linear.

HPV16 was detected in all HPV16 positive samples in all media tested. No HPV was detected in the HPV negative samples. The CN results for 2.4 × 10^5^ HPV16 copies per assay, the lowest HPV16 copy number tested, were similar in all media tested.

To further investigate the suitability of NHQ and NHQ-PT for HPV testing, the repeatability and reproducibility of results for HPV16 positive cells was evaluated. The HPV16 positive CN values for cells in NHQ and in NHQ-PT were highly repeatable with standard deviation of ≤ 0.5 CN for the 14 repeats of all HPV16 copy numbers tested (Table 1). CN results for NHQ and NHQ-PT samples with 10.0 × 10^5^ HPV16 copies per assay were comparable to those obtained in the accuracy experiment.

**Table 1:** Within-assay repeatability of HPV 16 samples in NHQ and NHQ-PT.

**Cell medium**	**Cells**	**Cells (×10**^**5**^**)/ assay**	**HPV 16 copies (×10**^**5**^**)/assay**^a^	**Mean CN**	**StdDev**
NHQ	Ca Ski	0.25	150.0	11.9	0.1
NHQ	SiHa	5	10.0	16.4	0.5
NHQ	SiHa	0.5	1.0	19.4	0.1
NHQ-PT^b^	Ca Ski	0.25	150.0	14.8	0.2
NHQ-PT	SiHa	5	10.0	17.9	0.4
NHQ-PT	SiHa	0.5	1.0	21.5	0.2

CN, cycle number; StdDev, Standard Deviation.

a Estimated number of HPV 16 DNA per cell is 2 in every SiHa cell and 600 in every Ca Ski cell.

b The Manufacturers Positive Control “other HR HPV” channel produced an error on this run, however the HPV 16, HPV 18 and Internal control channels were not affected.

42 repeat tests comprising 3 runs over 2 days using samples with estimated 1.0 × 10^5^ HPV16 copies in both NHQ and NHQ-PT showed high reproducibility with low standard deviation of ≤ 0.5 CN (Table 2). Reassuringly, CN results for NHQ and NHQ-PT samples with 1.0 × 10^5^ HPV16 copies per assay approximated to those obtained in the previous repeatability experiment.

**Table 2:** Reproducibility of HPV results for 1 × 10^5^ HPV 16 genomic copies^a^ in NHQ and NHQ-PT media.

**Media**	**Days, runs**	**Repeats**	**Mean CN**	**StdDev**
NHQ	2 days, 3 runs	42	19.8	0.2
NHQ-PT	2 days, 3 runs	42	21.4	0.3

CN, cycle number; StdDev, Standard Deviation.

a Estimated number of HPV 16 DNA per cell is 2 in every SiHa cell.

**Fig. 2:**
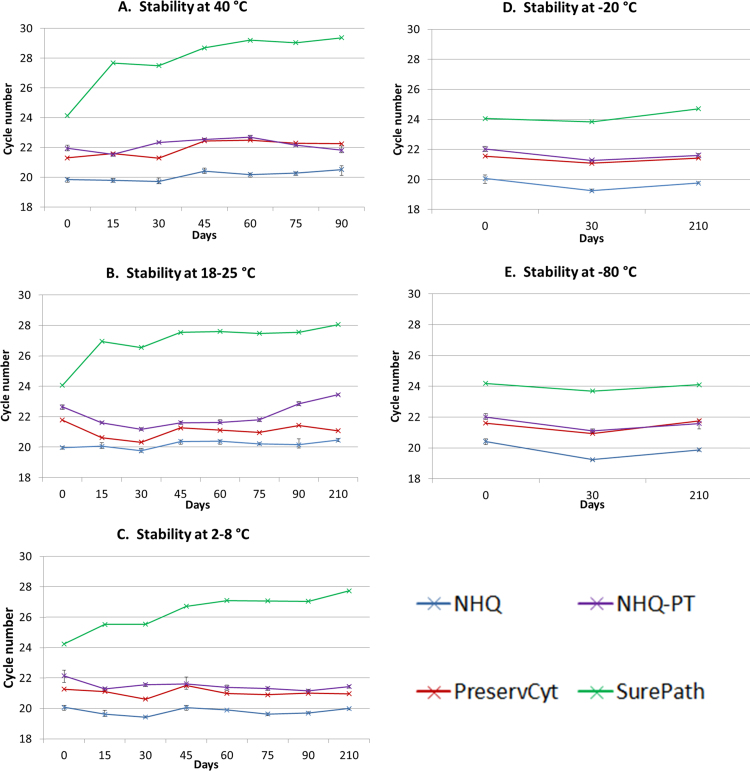
Stability of 1.25 × 10^5^ SiHa cells per ml with an estimated 2.5 × 10^5^ copies per ml of HPV 16 genomic DNA in 3 lots of NHQ, 3 lots of NHQ-PT, 1 lot of Preservcyt and 1 lot of Surepath media at A) 40 °C, B) 18–25 °C, C) 2–8 °C, D) −20 °C and E) −80 °C. NHQ, Novaprep HQ+ orange; NHQ-PT, Novaprep HQ+ Orange (pre-treated). Mean Ct and Min/max bars presented for the 3 lots of NHQ & NHQ-PT.

Stability of HPV 16 positive SiHa cells in the NHQ and NHQ-PT was compared to stability in Preservcyt (PS) and Surepath (SP), which are CE-marked for storage of samples for Realtime HPV testing (Fig. 2).

SiHa cells produced HPV16 detected results at each timepoint for all media, with stable HPV16 CN results. NHQ samples mimicked PS in that CN values remained stable across all temperatures tested. NHQ samples were stable for at least 210 days (seven months) at 18–25 °C, 2–8 °C, −20 °C and −80 °C; and 90 days at 40 °C. The CN values for NHQ samples were consistently around 2 CN lower than for PS, suggesting an improved recovery of DNA. Interestingly, NHQ-PT stability and CN values were almost identical to those for PS, with little variation in CN values in all storage conditions tested. NHQ-PT CN values did start to rise after 75 days (3.5 months) at 18–25 °C, suggesting stability of cells in NHQ-PT is less than that of NHQ and PS at this temperature. There was no discernible lot to lot variation for NHQ and NHQ-PT.

The largest variation in CN values was observed for SP at 18–25 °C and 40 °C, where an increase of > 2.85 CN from Day 0 was observed at the first timepoint, 15 days storage. A gradual CN increase was also observed for SP at 2–8 °C, especially over the first 60 days of storage. Only SP cells stored frozen at −20 °C or −80 °C produced stable CN values over seven months.

## 4 Conclusions

In this first stage validation and analytical study, suitability of NHQ and NHQ-PT storage and transportation media for automated HPV testing using the Abbott m2000 system was evaluated. In the context of HPV testing, the cell storage media is most likely to affect the integrity and recovery of DNA, rather than the performance of the HPV assay [10], [11], [12]. Cervical cell lines where HPV is integrated into the cell's genome were used as simulated samples instead of plasmids, which are often used as sample material in analytical studies. It was considered sufficient to include cells containing only a single HPV target, HPV16. The use of cell lines in preservatives is limiting due to the absence of potential interfering substances that might be experienced in clinical samples but gives a method of producing highly comparable numbers of cells and therefore DNA concentration between preservative types.

The impact of using NHQ or NHQ-PT on the accuracy of the HPV assay was evaluated in comparison to the manufacturer's own media. HPV16 results equivalent to ATB were obtained. There was some variation between different media, especially within the range of 4.8–20 × 10^5^ HPV16 copies per assay, where NHQ samples gave CN values up to 5 cycles lower than ATB. NHQ-PT CN values were between those for NHQ and ATB. This is unlikely to be due to inaccuracy in cell numbers as CN values for each duplicate, which were each prepared from different cell samples, had low standard deviation. A possible explanation is that cells in NHQ or NHQ-PT enable more efficient DNA recovery compared to ATB. However, this effect was not evident with the lowest cell numbers tested. It could be interesting to explore the performance of NHQ and NHQ-PT with copy numbers that produce CN values close to the assay's clinical cut-off of 32 cycles. Any impact of NHQ and NHQ-PT on CN values in this range could impact test performance. A study using NHQ and NHQ-PT could be performed to fully evaluate the suitability of NHQ and NHQ-PT for cervical screening using the Realtime HPV assay on clinical samples.

The stability of cervical cells for HPV testing is a critical factor for specimen preservation. In many clinical settings, short intervals between collection and controlled storage of samples are not feasible. Ideally, samples would remain stable for weeks or months at ambient temperature. Based on manufacturer's instructions, cervical samples in ATB are stable for 14 days at 2–30 °C, or 90 days at −10 °C or colder. Preservcyt claims stability of four months at 15–30 °C, and six months at 2–8 °C or below −10 °C. Surepath claims stability of two months at 15–30 °C, and six months at 2–8 °C or below −10 °C.

In this study, we showed that stability of simulated specimens in NHQ was comparable to Preservcyt: At least seven months at 18–25 °C, 2–8 °C, −20 °C or −80 °C; and at least three months at 40 °C. There was little variation in CN values over this time. This suggests that NHQ should provide long-term stability for clinical samples. Preservcyt samples have shown stability up to 2.5 years when stored at 2–8 °C, and as long as 8 years stored at ambient temperatures [15], [16]. NHQ has the potential to achieve similar performance.

Stability of cells in NHQ-PT was similar to NHQ, with the exception of a CN value increase at 18–25 °C after 3.5 months. This did not affect the result of the HPV test, but samples with a lower HPV16 copy number could be impacted after 3.5 months at this temperature. NHQ-PT CN values were higher than NHQ values and very close to Preservcyt. It is possible that the efficacy of DNA extraction from NHQ-PT is slightly less than that in NHQ, but similar to Preservcyt.

To our knowledge, this is the first study where stability of cervical cells for HPV testing was assessed in pre-treated LBC media. Results look promising, pre-treatment of NHQ and subsequent storage should not negatively affect the performance of the HPV test. However, a clinical study should be performed to confirm this.

NHQ and NHQ-PT showed improved stability over Surepath in terms of CN variation at all temperatures except −20 °C and −80 °C. CN values for Surepath samples stored at 18–25 °C or 40 °C increased by approximately three within the first 15 days of storage, even though the Realtime HPV assay product insert claims two months’ stability at 15–30 °C. This difference may be due to the fact that simulated samples were used to assess stability, whereas clinical samples may have been used to collate data for the Realtime HPV assay product insert. Surepath also produced the highest CN results on Day 0. Poor nucleotide recovery from Surepath samples has been previously reported, possibly due to fixation by the formalin included in this media [15], [17]. This effect was most apparent within the first 6 days, which concurs with our data. However, we did not perform testing between days 0 and 15 [17].

Stability was assessed at a DNA concentration much higher than the limit of detection and further studies are needed to assess the influence of preservatives at lower target level in any of the Preservative media tested here.

In conclusion, based on this first stage validation analytical study, preparation and storage of cervical cells in NHQ and pre-treated NHQ is suitable for the Abbott Realtime High Risk HPV assay. There should be no reservations in inclusion of NHQ, with or without manufacturer recommended pre-treatment, in any further validation clinical performance evaluation of this assay.

## Competing interests

Lab21 is a wholly owned subsidiary of Novacyt Group.

## Funding

This research was supported by a grant from Abbott Molecular.

This work was supported by Novacyt SA who provided reagents and consumables for the project.

This research did not receive any specific grant from funding agencies in the public, or not-for-profit sectors.

## Ethical approval

Not required.
